# Remote Psychological Interventions for Fear of Cancer Recurrence: Scoping Review

**DOI:** 10.2196/29745

**Published:** 2022-01-11

**Authors:** Clizia Cincidda, Silvia Francesca Maria Pizzoli, Gabriella Pravettoni

**Affiliations:** 1 Applied Research Division for Cognitive and Psychological Science European Institute of Oncology Scientific Institute for Research, Hospitalization and Healthcare (IRCCS) Milan Italy; 2 Department of Oncology and Hemato-Oncology University of Milan Milan Italy

**Keywords:** fear of cancer recurrence, cognitive behavioral therapy, acceptance and commitment therapy, mindfulness, eHealth, blended intervention

## Abstract

**Background:**

Patients with cancer and survivors may experience the fear of cancer recurrence (FCR), a preoccupation with the progression or recurrence of cancer. During the spread of COVID-19 in 2019, patients and survivors experienced increased levels of FCR. Hence, there is a greater need to identify effective evidence-based treatments to help people cope with FCR. Remotely delivered interventions might provide a valuable means to address FCR in patients with cancer.

**Objective:**

The aim of this study is to first discuss the available psychological interventions for FCR based on traditional cognitive behavioral therapies (CBTs) or contemporary CBTs, in particular, mindfulness and acceptance and commitment therapy, and then propose a possible approach based on the retrieved literature.

**Methods:**

We searched key electronic databases to identify studies that evaluated the effect of psychological interventions such as CBT on FCR among patients with cancer and survivors.

**Results:**

Current evidence suggests that face-to-face psychological interventions for FCR are feasible, acceptable, and efficacious for managing FCR. However, there are no specific data on the interventions that are most effective when delivered remotely.

**Conclusions:**

CBT interventions can be efficacious in managing FCR, especially at posttreatment, regardless of whether it is delivered face to face, on the web, or using a blended approach. To date, no study has simultaneously compared the effectiveness of face-to-face, web-based, and blended interventions. On the basis of the retrieved evidence, we propose the hypothetical program of an intervention for FCR based on both traditional CBT and contemporary CBT, named Change Of Recurrence, which aims to improve the management of FCR in patients with cancer and survivors.

## Introduction

### Fear of Cancer Recurrence

Along the trajectory of cancer care, which in recent years has been extended because of new technologies [1], patients and survivors might experience fear of cancer recurrence (FCR), which is the concern that cancer will progress when it is stable or that it may return after the end of treatment [2]. Low levels of FCR could be adaptive for patients and survivors, as they promote and encourage the maintenance of medical follow-up, engagement in healthy lifestyle changes, and greater attention to signs of new or recurring cancer [3]. However, when FCR becomes distressing, patients can engage in negative health behaviors (eg, overusing health services and avoiding appropriate tests to identify cancer recurrence) and experience higher psychological distress and poorer quality of life (QoL) [3-5]. In addition, significant relationships emerged between FCR and certain sociodemographic variables (eg, younger age and having young children) and psychological factors (eg, anxiety and depression), whereas conflicting data emerged regarding the characteristics of tumor and cancer treatments [3]. With the advent of emerging nanomaterial-based approaches that would promote early diagnosis [6], these could favor better treatment efficacy and a reduction in FCR. However, this is an innovative area that is yet to be explored.

Currently, patients with cancer and survivors experience higher levels of loneliness, FCR, anxiety, and depression because of the diffusion of COVID‐19. Indeed, the pandemic forced people to social distance and caused several challenges for patients with cancer and survivors, such as maintaining social support and continuing their cancer treatment or medical check-ups regularly [7-10]. Despite the current situation, the need to identify effective evidence-based treatments to help people cope with FCR has increased in the past decade. In clinical settings, clinicians can help patients and survivors using psychological support, psychoeducational interventions, or psychotherapy. Data from recent systematic reviews and a meta-analysis of 23 controlled trials have shown that psychological interventions for FCR are feasible, acceptable, and efficacious for managing FCR, having a small-to-medium but a robust effect that persists at postintervention (Hall et al [11]: Hedge *g*=−0.36; Tauber et al [5]: Hedge *g*=0.33) and follow-up (Tauber et al [5]: Hedge *g*=0.30) [4,12]. Interestingly, in these reviews, most psychological interventions were based on traditional cognitive behavioral therapies (CBTs) or contemporary CBTs (eg, mindfulness and acceptance and commitment therapies [ACTs]). The first, traditional CBTs, focused on the contents of thoughts and aimed to identify and modify people’s negative thoughts or biases to reduce dysfunctional emotions and promote psychological adjustment, whereas the second, contemporary CBTs, focused on mental processes and aimed to modify how people relate to their inner experiences [3-5,11,12]. In particular, Tauber et al [5] found that FCR symptoms were more responsive to contemporary CBTs (Hedge *g*=0.42) compared with traditional CBT (Hedge *g*=0.24) at postintervention; however, this greater effect did not persist over time [5]. Considering the existence of several declinations of CBT approaches, it is important to understand the interventions that are effective in helping patients with cancer with decreasing FCR. As the pandemic forced patients with cancer into obliged isolation, a critical analysis of the different modalities in which these types of therapies can be delivered (eg, face to face or via remote and web-based approaches) might be useful. Indeed, apart from traditional in-presence interventions, blended (a mixed method comprising both web-based and face-to-face therapy) or remote care for patients with cancer has been increasingly applied [13]. Furthermore, during the pandemic, the management of patients with cancer has raised medical issues [14,15] and psychological issues [16].

### Objective

In this paper, we aim to critically revise and systematize the available evidence on the effectiveness of different modalities and approaches of CBT psychological interventions for FCR. Specifically, this review aims to summarize studies dealing with face-to-face, remote, and blended interventions based on traditional CBTs or contemporary CBTs, in particular, mindfulness and ACT, used to reduce FCR. Finally, we propose a possible program based on the retrieved literature.

## Methods

Electronic searches were performed using PubMed, MEDLINE, and Embase between November and December 2020, with no time limits. Original articles were considered in English, Italian, or Spanish languages, with participants aged ≥18 years. Keywords searched in titles and abstracts included *fear of cancer recurrence* combined with terms such as *cognitive behavioral therapy*, *acceptance and commitment therapy*, *mindfulness*, *mindfulness-based stress reduction*, *blended therapy*, *face-to-face intervention*, and *online intervention*. The reference lists in the relevant systematic reviews were manually searched for additional contributions that met our inclusion criteria. The search was limited to only full-text articles. The studies included in this paper met the following main criteria: (1) articles dealing with patients with cancer with FCR, (2) articles including traditional or contemporary cognitive psychological interventions for FCR (CBT, ACT, and mindfulness), and (3) articles presenting quantitative data or study protocols. Commentaries, editorials, and conference proceedings were excluded. Research papers on traditional and contemporary CBT interventions (classical CBT, mindfulness, and ACT) were included.

Initially, the search strategy yielded 470 articles that were screened for irrelevant or duplicate articles. The remaining articles were assessed and selected by screening the abstracts, followed by full-text reading and selection according to the predefined inclusion and exclusion criteria. Of the 470 articles, 35 (7.4%) articles that focused on psychological interventions for reducing FCR were selected. The results were organized according to the modality of delivery of the therapy (face to face, remote, and blended).

## Results

### Face-to-face Traditional CBT, Mindfulness, and ACT

Psychological interventions for patients with cancer and survivors based on the principles of traditional CBT aim to encourage patients to identify, express, and deal with their fears and emotional reactions related to cancer and improve their ability to cope with them to maintain their QoL and evaluate and alter life priorities [17-23]. Traditional CBT interventions can be delivered in groups [17,19,21,22,24] or individually [18,20,23,25] and have short-term benefits [17,19,22-24] and significant long-term effects on FCR [17,18,20].

Clinicians often begin the interventions with psychoeducation on FCR to explain what it is and how it presents and maintains during everyday life [22,23]. Moreover, through these interventions, patients can improve their problem-solving skills and use of personal and social resources, enhance their self-esteem and autonomy [20,21], and look into their illness beliefs and behaviors [18]. Specifically, patients address irrational thoughts through cognitive reframing to reduce catastrophic interpretations of physical symptoms and emotions [19,21-23]. Some examples of psychological interventions are Side by Side, a couple of skill interventions proposed by Heinrichs et al [24], and the adjustment to the fear, threat or expectation of recurrence intervention theorized by Humphris and Rogers [18]. In particular, the Side by Side program takes place at the couple’s home and comprises 4 biweekly face-to-face sessions with a therapist, focused on communication skills and dyadic coping [24]. Instead, the adjustment to the fear, threat or expectation of recurrence intervention is for patients—the caregiver can only be included if desired by the patient—and is intended to encourage patients to express and process fears related to FCR and explore illness beliefs and behaviors. During the sessions, patients also practiced relaxation exercises [18].

Psychological interventions based on mindfulness, which is a particular way to pay attention to the present moment without judgment, ensure that people turn away from unhealthy beliefs, thoughts, or emotions, maintaining awareness of the present moment [26]. Clinicians can choose between different mindfulness-based programs, such as mindfulness-based stress reduction (MBSR), mindful movement program, or mindfulness-based cognitive therapy (MBCT) [4,5,27]. The MBSR program aims to self-regulate arousal in response to stressful situations or symptoms and reduce the intensity of cognitive processes by lowering the frequency of negative automatic thinking [4,27]. This program comprises 1-hour session conducted by a psychologist and home practice following a manual and audiotapes (eg, sitting meditation, walking meditation, body scan, and yoga), with data showing that it has a significant effect on FCR in people with cancer at postintervention [28-32]. Instead, the mindful movement program combines mindfulness with self-directed movement for patients to explore and understand their thoughts, feelings, and sensations. Data report a significant effect on FCR at 12 weeks after treatment; however, this effect does not maintain at 18 weeks after treatment [33]. The MBCT program aims to teach participants to be more mindful in daily life through meditation exercises, yoga, group discussions, and didactic teaching. This program comprises 8 weekly sessions, a silent day, and daily homework. Compared with usual care, patients who receive this program report significantly lower levels of FCR (Cohen *d*=0.27) [27].

Finally, ACT interventions explain psychological distress through psychological inflexibility, a construct that comprises behaving under the strict control of rigid personal thoughts, feelings, and other internal experiences [34]. ACT aims to reduce psychological inflexibility, limit the use of maladaptive coping strategies, and enhance psychological flexibility, which lets people live mindfully according to their values and accept both negative and positive events [34]. However, in the literature, there were only 9% (3/35) of studies that applied ACT to FCR [35-37], and only 3% (1/35) was a randomized controlled trial (RCT) assessing the feasibility and preliminary efficacy of ACT interventions for FCR at postintervention and over time [36]. Johns et al [36] proposed a group intervention based on ACT principles, which aimed to promote adaptive coping through acceptance, cognitive defusion, awareness, and perspective-taking exercises while supporting survivors of breast cancer (BC) in aligning their behavior with personal values to cope with fears. Furthermore, participants improved the skills learned during the session by performing home awareness practice [36]. Arch and Mitchell [35] developed a group manual and workbook for participants comprising experiential exercises, metaphors, discussions, and homework. This was aimed at helping participants to be aware of and accept thoughts and emotions about cancer, eliminate rigid thoughts and beliefs about cancer and themselves through psychological flexibility, and define personal values and commit to pursuing meaningful activities in line with those values [35]. All these interventions suggest that ACT intervention could be useful for reducing FCR [35-37]. Moreover, in the literature, a manualized intervention, called ConquerFear, focuses on reducing the impact of FCR based on metacognitive therapy, the Common Sense Model of illness, the Self-Regulation of Executive Function Model, and relational frame theory, which form the theoretical basis for ACT (more details are provided in Butow et al [38]). In particular, this intervention comprises 5 face-to-face sessions, each of which is associated with home exercises on the skills learned in the session. Patients, through the ConquerFear intervention, learned new strategies to control worry, modified dysfunctional beliefs related to worry, acquired appropriate monitoring and screening behaviors, learned to accept the uncertainty caused by a cancer diagnosis, and defined values and goals based on them. This intervention reduced FCR severity at posttreatment and over time [39,40]. A summary of the described face-to-face psychological interventions for FCR is provided in Table 1.

**Table 1:** Face-to-face psychological interventions.

Therapy and authors			Cancer		Study design		Intervention and groups		Results
**Traditional CBT ^a^**									
	Heinrichs et al [24]	BC^b^ or GC^c^		RCT^d^		Side by Side: 4 biweekly couple skills sessions Couples Control Program		Side by Side had a greater effect on FoP^e^ than the Couples Control Program; however, this difference disappeared by 16 months after the diagnosis.	
	Herschebach et al [17]	Different type		Longitudinal study		4 session cognitive behavioral group therapy Supportive experimental group therapy UC^f^		FoP decreased significantly over time in both intervention groups in contrast to the control group that showed only short-term improvements.	
	Humphris and Rogers [18]	Different type		RCT		AFTER^g^: 6 weekly sessions of traditional CBT individual therapy UC		AFTER intervention improves FCR^h^ only at the immediate short-term follow-up (MWU^i^: z=2.06; *P*=.04).	
	Lebel et al [19]	Survivors of BC or ovarian cancer		A single-arm multisite study		6-week cognitive-existential group intervention		Significant reductions of FCR levels immediately after it and at the 3-month follow-up	
	Manne et al [20]	GC		RCT		CCI^j^: 7 weekly sessions of individual therapy and 1 telephone session 2 or 3 weeks after session 7 A supportive counseling intervention: 7 weekly sessions of individual therapy and 1 telephone session 2 or 3 weeks after session 7 UC		CCI did not affect FCR.	
	Savard et al [22]	Different type		Development and feasibility		4 weekly group CBT sessions		Significant reductions of FCR levels	
	Tomei et al [23]	Different type		RCT pilot		FCR intervention: 6-week sessions		Significant reduction of FCR levels at postintervention and at 3-month follow-up	
**Contemporary CBT**									
	Crane-Okada et al [33]	Survivors of BC		RCT pilot		12-week mindful movement program intervention		Significant effect on FCR at 12 weeks posttreatment; however, this effect does not maintain at 18 weeks after treatment	
	Lengacher et al [28]	Survivors of BC		RCT		6‐week MBSR^k^ program UC		MBSR reduces FCR more than usual care (11.6 vs 9.3) at 6 weeks.	
	Lengacher et al [29]	Survivors of BC		Feasibility of the intervention		8-week MBSR program		Significant effect on FCR	
	Lengacher et al [30]	BC		A single-arm multisite study		6-week MBSR (BC) program UC		MBSR (BC) reduces FCR; MBSR (BC) compared with UC had a favorable change in FCR problems that mediated the effect of MBSR (BC) on 6-week change in perceived stress (z=2.12; *P*=.03) and state anxiety (z=2.03; *P*=.04)	
	Lengacher et al [31]	Survivors of BC		RCT		2-hour sessions once per week for 6 weeks of an MBSR (BC) UC		Significant improvement of FCR in the MBSR (BC) group compared with usual care	
	Victorson et al [32]	PC^l^		RCT pilot		8‐week MBSR intervention An attention control arm		MBSR significantly reduces PC anxiety and uncertainty intolerance	
	Arch and Mitchell [35]	Survivors of BC		Pilot study		7 weekly 2-hour sessions of ACT^m^		FCR decreases through 1 week following the last group session (post; Cohen *d*=0.34; *P*<.05) and 3 months following post (Cohen *d*=0.66; *P*=.001) but not during the month-long baseline period (Cohen *d*=0.11; *P*=.43)	
	Johns et al [36]	Survivors of BC		RCT		6 weekly 2‐hour group sessions of ACT 6 weekly 2‐hour group sessions of SE^n^ One 30‐minute group coaching session (EUC^o^)		SE and ACT reduce FCR severity over time; however, only ACT produced significant reductions at each time point relative to baseline, with between-group differences at time point 4 substantially favoring ACT over SE (Cohen *d*=0.80; *P*<.001) and EUC (Cohen *d*=0.61; *P*<.01)	
	Marín and Soriano [37]	BC		Open trial		1 session of ACT Waiting list		Defusion contributes to decreasing FCR, and this effect is maintained 3 months after the intervention.	
	Butow et al [38,39]	BC or CRC^p^; BC or CRC or melanoma		Study protocol of an RCT; RCT		ConquerFear intervention: 5 face-to-face sessions over 10 weeks Taking-it-Easy relaxation therapy: 5 face-to-face sessions over 10 weeks		ConquerFear is efficacy compared with attention control in reduction of FCR immediately after therapy and 3 and 6 months later	
	Smith et al [40]	BC		Pilot study		ConquerFear intervention: 5 face-to-face sessions over 10 weeks		ConquerFear is feasible, acceptable, and shows potential efficacy for FCR	

^a^CBT: cognitive behavioral therapy.

^b^BC: breast cancer.

^c^GC: gynecologic cancer.

^d^RCT: randomized controlled trial.

^e^FoP: Fear of Progression.

^f^UC: usual care.

^g^AFTER: adjustment to the fear, threat or expectation of recurrence.

^h^FCR: fear of cancer recurrence.

^i^MWU: Mann-Whitney *U* test.

^j^CCI: coping and communication-enhancing intervention.

^k^MBSR: mindfulness‐based stress reduction.

^l^PC: prostate cancer.

^m^ACT: acceptance and commitment therapy.

^n^SE: survivorship education.

^o^EUC: Enhanced Usual Care.

^p^CRC: colorectal cancer.

All these approaches, which are delivered face-to-face, present some criticisms: they were relatively expensive and time and resource intensive, and patients could be reluctant to return to the hospital where cancer treatment took place [41]. In the current period, such limits might be overcome by remotely delivered internet-based interventions.

### Traditional CBT, Mindfulness, and ACT Delivered Through eHealth

Over the past decades, owing to the increasing use of new technologies for the treatment of psychological aspects, clinicians have applied remotely delivered psychological techniques in the field of mental health and health care settings, giving rise to eHealth [42]. Web-based interventions overcame the criticisms of face-to-face interventions, as they can be performed from patients’ homes, even for those who are in remotely located places or have reduced mobility for health issues [43,44]. Moreover, eHealth reduces health care costs, as it requires few economical resources to be allocated for personnel needed compared with the need for personnel’s engagement in face-to-face therapies [45]. Finally, eHealth also offers the chance for self-management and continuity of care [46]. However, there are some disadvantages related to eHealth interventions, such as the lack or reduction of personal interaction, poor adherence, and less engagement [47].

Considering the clinical target, eHealth proved to be effective in the field of mental health and psychological treatments [42,48], whereas in specific oncological settings, it has limited and inconsistent findings [49,50].

eHealth based on CBT involves the delivery of clinical CBT content via the internet and provides content in several formats, for example, text, video, and audio files and interactive elements.

Regarding traditional CBT, Lichtenthal and al [51] proposed a home-delivered cognitive bias modification intervention to reduce FCR. The intervention, Attention and Interpretation Modification for Fear of Breast Cancer Recurrence (AIM-FBCR), targets 2 types of cognitive biases: attentional bias, which is assessed through a modified dot-probe task, and interpretation bias, which is assessed through a word–sentence association paradigm [51]. In a preliminary pilot randomized controlled study (n=110), participants completed 8 personalized treatment sessions twice a week for 4 weeks, and the results showed that this intervention was effective in reducing health worries in patients with cancer [51]. Another example is the web-based self-help training performed by van Helmondt et al [52]. The program, Cancer Recurrence Self‐Help Training trial, comprises a psychoeducation model and a model based on the basic principles of CBT, and 4 optional modules (rumination, action, relaxation, and reassurance) to choose from. Each module comprises an instructive part (texts, videos, or audio files) and a part made of exercises. Patients could be supported on the web personally by emailing a coach [52]. However, the same authors found that there was no effect of this intervention on FCR [44].

Regarding contemporary CBT, specifically for the concerns regarding eHealth mindfulness-based programs used alone in the cancer settings, evidence related to FCR is scarce. However, the internet-based MBCT (eMBCT) intervention, which is a combination of MBCT and CBT, demonstrated reductions in FCR (Cohen *d*=0.53) and rumination and improvements in mental health–related QoL compared with standard care [27]. Patients accessed this intervention, which was delivered individually, through a secure website in which they found an introduction module and daily meditation exercises with meditation audio files. During the intervention, the patients had to fill out practice diaries. The intervention lasted 8 weeks and included a silent day, at the end of which patients were asked to write about their experiences in an essay and send it to the therapist, who returned written feedback [27]. On the basis of MBSR, there is a mobile MBSR for BC program proposed by Lengacher et al [53]. Patients accessed this program via an iPad after having received a user manual and orientation on how to use the iPad. The content of the program comprised video files on formal meditative techniques (eg, sitting meditation, walking meditation, body scan, and gentle Hatha yoga) and audio files on informal meditative techniques (eg, integrating mindfulness into daily life activities). Moreover, participants could learn the formal and informal meditative techniques provided on the iPad through a physical manual. The results of a pilot study on this program showed that it might be feasible and acceptable, improving FCR scores [53].

Finally, with regard to web-based interventions based on ACT, the studies recovered in the literature were few, and in the oncology field, we found only 11% (4/35) of studies; 50% (2/4) of them were for patients with cancer and the others for partners [54-57]. One of the studies explored the acceptability, feasibility, efficacy, and cost-effectiveness of an internet intervention for survivors of BC called iNNOVBC [57]. To date, the authors have presented only the protocol of this intervention, which comprises 10 modules, 5 of which are optional. In the mandatory modules, there was 1 about anxiety, worries, and fear of recurrence; for that reason, we presented this program. The key components were psychoeducation; 4 of the 6 core processes of psychological flexibility (acceptance, cognitive defusion, values, and committed action); behavioral activation; and relaxation that patients learned through experiential exercises, metaphors, and homework. During therapy, patients could communicate with a therapist via SMS text messaging, chat, email, and videoconference [57].

Finally, in the literature, we found examples of web-based interventions based on multiple theories, such as e-TC and iConquerFear [49,58]. The e-TC includes 6 interactive modules that take approximately an hour to complete. The therapeutic contents were based on traditional CBT, ACT, metacognitive therapy, mindfulness, and relaxation. Patients were trained to restructure or accept their unpleasant thoughts and feelings and cope with stressful situations [49]. The results of the pilot tests showed that the participants were highly satisfied with the program, although they suggested that limited time for men was an obstacle to using and completing the program, and men with a more recent diagnosis and a level of higher distress may be more likely to commit to the program. However, e-TC appears to be a feasible and acceptable web-based intervention for survivors of testicular cancer [49]. To date, no data have supported the effectiveness of this intervention. Moreover, the iConquerFear intervention, based on the aforementioned ConquerFear therapy manual [38], comprises 6 modules, 5 therapeutic and 1 as the introduction, including audio, video, and text contents. Patients completed the modules in 1 to 2 hours over 1 to 2 weeks, and between the modules, they practiced with the skills learned [58]. However, no data supported the effectiveness of this intervention, although there is only a qualitative evaluation of the usability of iConquerFear [58].

A summary of the aforementioned studies on psychological interventions delivered through eHealth is provided in Table 2.

**Table 2:** Remote psychological interventions.

Therapy and authors		Cancer	Study design	Intervention	Results
**Traditional CBT^a^**					
	Lichtenthal et al [51]	BC^b^	RCT^c^ pilot	AIM‐FBCR^d^: 8 personalized treatment sessions of 30 minutes each administered twice a week for 4 weeks A control condition program	The results of the current pilot study suggest the promise of AIM‐FBCR in reducing FCR^e^ in survivors of BC
	van Helmondt et al [44,52]	Survivors of BC	Study protocol; RCT	The Cancer Recurrence Self‐help Training trial: less fear after cancer—a tailored web-based self-help training (2 basic modules and 4 optional modules) UC^f^	There was no effect of the CBT‐based web-based self‐help training *Less fear after cancer* on FCR in the study
**Contemporary CBT**					
	Compen et al [27]	Different type of cancer	RCT	Individual internet-based MBCT^g^: access to a secure website containing material for 8 weeks plus a silent day and an inbox+weekly asynchronous written interaction with a therapist over email MBCT: 8 weekly 2.5-hour group sessions, a 6-hour silent day, and daily home practice assignments guided by audio files UC	Compared with UC, both interventions reduced FCR
	Lengacher et al [53]	BC	Feasibility	mMBSR (BC)^h^: sitting and walking meditation, body scan, and yoga and is designed to deliver weekly 2‐hour sessions for 6 weeks using an iPad	There was a significant improvement from baseline to 6 weeks after mMBSR (BC) in FCR
	Mendes-Santos et al [57]	Survivors of BC	Study protocol	iNNOVBC^i^: a 10-week guided internet-delivered individually tailored ACT^j^ influenced CBT intervention UC	Not yet available
	Heiniger et al [49]	TC^k^	Pilot study	e-TC: 6 interactive modules for 10 weeks	e-TC appeared to be a feasible and acceptable web-based intervention for survivors of TC
	Smith et al [58]	Different type of cancer	Study protocol	iConquerFear: 5 therapeutic modules completed in 1 to 2 hours over 1 to 2 weeks	Not yet available

^a^CBT: cognitive behavioral therapy.

^b^BC: breast cancer.

^c^RCT: randomized controlled trial.

^d^AIM‐FBCR: Attention and Interpretation Modification for Fear of Breast Cancer Recurrence.

^e^FCR: fear of cancer recurrence.

^f^UC: usual care.

^g^MBCT: mindfulness-based cognitive therapy.

^h^mMBSR (BC): mobile mindfulness‐based stress reduction for breast cancer.

^i^iNNOVBC: a guided internet-delivered individually tailored acceptance and commitment therapy–influenced cognitive behavioral intervention to improve psychosocial outcomes in breast cancer survivors.

^j^ACT: acceptance and commitment therapy.

^k^TC: testicular cancer.

### Traditional CBT, Mindfulness, and ACT Through Blended Care

Currently, in the field of eHealth treatments, the use of blended care is gaining rising visibility. Blended treatment or blended care are defined in literature as “technology-supported care,” with the term *blended* describing a combination of web-based and offline elements inside the same care-flow intervention. Generally, the offline part corresponds to face-to-face sessions, whereas for the web-based element, patients accessed a website or workbook, which facilitated further skill acquisition and learning [41]. To date, a single definition of what blended intervention exactly reserves was absent, as blended care is currently built with different approaches across studies [48].

In blended treatments, web-based and offline components are not standalone treatment pathways but rather interrelated methods that are strategically combined to build an intervention that merges the potential benefits of the 2 approaches [48,59]. In particular, blended care shares with eHealth the advantage of being flexible in application, having good accessibility, and saving travel time and costs [59,60]. It shares with face-to-face interventions the benefit of having a therapist as a guide, which increases adherence, prevents dropout, facilitates increased treatment intensity, and leads to better results than unguided treatments [61]. To date, the literature has demonstrated that blended therapy displays encouraging effects [48,62] and has shown efficacy in reducing FCR among early-stage survivors of cancer [63]. CBT was the first approach that was used to build blended interventions in the field of psychological health [64], emerging as an evolution of the classical CBT method and the method that was internet-delivered. Indeed, blended care CBT treatments (BC-CBTs) blend classical therapist-led CBT sessions with internet-delivered CBT components, and it constitutes an integrated therapy, putting together the gains of both approaches while relieving the limits of the single approaches. The interactive presence of the therapist, for instance, allows for the creation of a therapeutic alliance, which in turn is associated with higher motivation to begin and maintain commitment in care [65,66]. Furthermore, the presence of a therapist allows for a fair degree of personalization of treatment plans. Specifically, the therapist using BC-CBT can decide the extent to which face-to-face sessions, live video-based sessions with interactions between therapist and client, and remote digital care tools (lessons and exercises) that can be accessed by the client between sessions can be combined [67,68]. Moreover, internet CBT with the therapist’s support leads to better clinical outcomes, more stable results in time, and higher adherence compared with unsupported ones (eHealth) [69,70].

Considering traditional CBT, the Survivors’ Worries of Recurrent Disease study is an example of a BC-CBT intervention that combines traditional CBT, which is delivered face-to-face, with web-based activities (or workbook activities) [41,63,71]. This intervention aims to modify the cognitions and behaviors that maintain a high FCR through cognitive reframing, exposure and response prevention, psychoeducation, mindfulness, and relaxation exercises. It includes 5 face-to-face therapy sessions and 3 web-based or telephone sessions with a trained therapist. Participants could access a supportive website or workbook along with the therapy. During the first session, participants conceptualized their personal FCR model, which guided the course of the therapy. Subsequent sessions focused on acceptance, cognitive restructuring, and behavior modification. The final sessions consolidated the progress made by the patient and established a relapse prevention plan. Participants did homework to improve the skills learned during therapy. In an RCT of 88 survivors of cancer with high levels of FCR, participants who received this intervention had significantly less FCR compared with those who were allocated to the standard care group. The effect size was moderate to large (Cohen *d*=0.76), and both the clinical and self-reported improvements in FCR were higher in the BC-CBT group compared with the control group [63]. Finally, a recent study showed that the Survivors’ Worries of Recurrent Disease intervention was effective in reducing FCR compared with treatment as usual in the long term (mean difference −1.787, 95% CI −3.251 to −0.323; *P*=.02 at 15 months follow-up) [47].

Another example is the blended care for FCR study proposed by Luigjes-Huizer et al [72]. This intervention was developed to be delivered by primary care and not by professionals and institutes specializing in psycho-oncology, as the role of primary care in cancer and survival care is increasing. In particular, general practitioners and mental health workers provided an intervention specifically designed for FCR based on CBT, clinical experience, and input from patients, with web-based modules that focused on normalization, psychoeducation, and self-management. There were 2 modules based on CBT and 5 optional modules (eg, rumination, avoidance, relaxing, reassuring, and undertaking activities). The authors are conducting a 2-armed cluster-randomized trial to evaluate the effectiveness of this intervention [72].

In literature, there is another blended intervention based on traditional CBT, the colorectal cancer distress reduction intervention, which was proposed for survivors of colorectal cancer, which was not specific for FCR [73]. However, we propose this intervention as it comprises 3 separate modules, each of which deals with a specific ailment that the patient may experience, namely distress because of physical consequences, anxiety and FCR, and depression. It aims to facilitate adjustment and coping, reduce distress, and modify cognitions and behaviors. It lasted 14 weeks and comprised 5 individual sessions and 3 telephone consultations, combined with the use of the interactive self-management website, where patients found the homework. Patients discussed their homework completed on the website with a therapist during the individual sessions. In the first session, patients reported their cancer follow-up experiences with a focus on treatment goals, the distress they are experiencing, and their own unmet needs. During the second session, the therapist introduced and explained the basic skills of the CBT applied to the first module. The following sessions included psychoeducation, cognitive restructuring, behavior modification, and relaxation. In the last 2 sessions, the therapist and patient evaluated the reduction of distress and discussed the consolidation of long-term skills. The website included a general introduction module comprising 2 web-based homework sessions focused on introducing CBT and identifying personal goals. A total of 3 specific modules, including different types of self-management activities (eg, psychoeducational scripts, assignments tasks, screening tests, audio clips, and peer videos) and a general closing module focused on goal evaluation and relapse prevention. A 2-arm multicenter RCT of 160 survivors of colorectal cancer with high levels of ailment was initiated by the authors [73]. However, a cognitive behavior therapist decided to treat a 74-year-old male survivor of colorectal cancer with this intervention for 4 months and reported an improvement in psychological distress after intervention [74].

Regarding blended mindfulness or ACT-based interventions, there have been studies. On the basis of the aforementioned ConquerFear therapy, researchers want to test the efficacy in the short- and long-terms of a therapist-guided version of iConquerFear in reducing FCR and improving QoL in survivors of colorectal cancer. This intervention differs from iConquerFear because of the presence of the therapist through a messenger function with whom patients can communicate asynchronously. The therapist had the role of a motivator and coach, answering the questions and giving feedback on the exercises and written material [75]. A summary of the described studies is presented in Table 3.

**Table 3:** Blended psychological interventions.

Therapy and authors		Cancer	Study design	Intervention	Results
**Traditional CBT^a^**					
	van de Wal et al [47,63,71]	BC^b^, PC^c^, or colorectal; survivors; BC, PC, and CRC^d^	Study protocol; RCT^e^; RCT	The SWORD^f^ study: 5 individuals 1-hour F2F^g^ sessions+three 15-minute web-based sessions based on traditional CBT; UC^h^	SWORD had a greater effect on FCR^i^ than UC with a moderate-to-large effect size (Cohen d=0.76). SWORD had a greater effect on FCR than UC (mean difference −1.787, 95% CI −3.251 to − 0.323; *P*=.02) at the 15-month follow-up.
	van de Wal et al [41]	Survivors of BC	Case study	The SWORD study: 7 F2F therapy sessions and 1 telephone session based on traditional CBT	CBT reduced FCR over time (last follow-up at 12 months after therapy).
	Luigjes-Huizer et al [72]	—^j^	Study protocol	BLANKET^k^: 2 CBT modules+5 optional modules; UC	—
	Leermakers et al [73]	Survivors of CRC	Study protocol	CORRECT^l^: 5 F2F sessions +3 telephone sessions and an interactive self-management website; UC	—
	Döking et al [74]	Survivors of CRC	Case study	CORRECT for 4 months: 5 F2F+3 telephone sessions and an interactive self-management website	The intervention was successful in reducing the distress of a survivor of cancer.
**Contemporary CBT**					
	Lyhne et al [75]	Survivors of CRC	Study protocol	Therapist-guided iConquerFear: 5 modules; UC	—

^a^CBT: cognitive behavioral therapy.

^b^BC: breast cancer.

^c^PC: prostate cancer.

^d^CRC: colorectal cancer.

^e^RCT: randomized controlled trial.

^f^SWORD: Survivors’ Worries of Recurrent Disease.

^g^F2F: face to face.

^h^UC: usual care.

^i^FCR: fear of cancer recurrence.

^j^The type of cancer was not specified.

^k^BLANKET: blended care for fear of cancer recurrence.

^l^CORRECT: colorectal cancer distress reduction.

## Discussion

### Principal Findings

On the basis of the data presented in the literature, we could infer that a psychological intervention based on CBT is efficacious in managing FCR, especially at posttreatment [4,5,11,12]. Taking into consideration the results of traditional CBT, almost all psychological interventions reduced FCR at postintervention and follow-up regardless of whether they were delivered face to face, on the web, or blended [17,19,41,51,63,71]. Only 1 study did not show any effect on FCR [44]. The remaining studies reduced the level of FCR only at posttreatment [18,20,22-24]. Moreover, there was a lack of data on the efficacy of 2 blended interventions based on traditional CBT [72,73]. To date, no study has simultaneously compared the effectiveness of face-to-face, web-based, and blended interventions.

Regarding psychological interventions based on mindfulness, only 1 RCT study compared MBCT and eMBCT with usual care and found that MBCT and eMBCT were equally efficacious compared with treatment as usual in reducing FCR [27,76,77]. In general, face-to-face and web-based interventions reduced FCR at posttreatment [28-33,53]. However, there was a lack of studies on blended interventions based on mindfulness.

The retrieved studies in the literature on ACT reported only protocols and feasibility or qualitative studies, with no quantitative data on the effectiveness of such an approach, except for 2 RCTs—1 that provided a face-to-face intervention and the other 1 a web-based intervention [35-37,57]. However, little data on the feasibility of this approach have yielded promising results [56]. Even for ACT, no studies used blended interventions.

Finally, there were 2 programs based on multiple theories: ConquerFear, which clinicians could use face to face, via the web, or as blended; and e-TC. However, there was only quantitative data supporting the efficacy of face-to-face ConquerFear, which had a short and long-time effect on FCR [39,40].

Hence, it was difficult to decide which one to choose. If for interventions based on traditional CBT, there were more data, for the contemporary CBT, the information would still be limited, even more so if we take into consideration the new methods of administering the interventions. Although it is normal given the youth of these new modalities, the need to find effective web-based or blended treatments is increasingly urgent. Furthermore, when it comes to clinical applications, the choice of what should be interactive is not trivial. Indeed, it has been shown that therapists believe that the complexity of patients’ problems requires tailored blended treatment. It has also been found that therapists and patients have different points of view regarding what components of the therapy they would prefer to be presented in a web-based-remote way [59]. The parts of treatment that can best be offered on the web or face to face can differ among patients (based on ability, preference, severity, and type of problems) and should thus be considered for each patient individually [59]. For that reason, further studies should explore patients’ preferences, not only about the content of the intervention but also the type of intervention (face-to-face, web-based, or blended) they prefer. Another point that is currently lacking in the retrieved literature concerns the stigma of mental health care [78] and how different modalities of delivery of treatments might address this issue. Indeed, mental health stigma has been found to have a small- to moderate-sized negative effect on help-seeking behaviors [79]. Interestingly, web-based interventions might be specifically built to target mental health stigma [80].

Patients and survivors might benefit from blended interventions as they have the potential benefits of face-to-face and web-based approaches. In particular, through a blended intervention, patients might maintain the therapeutic alliance with the therapist without the necessity of meeting them weekly because of, for example, a website in which they have to do some homework to practice the skills learned during the face-to-face session. We believe that an integrative intervention based on both traditional CBT and contemporary CBT would be the best choice.

### Future Directions: “Change of Recurrence”

#### Overview

On the basis of the retrieved evidence, we propose the hypothetical program of an intervention for FCR based on both traditional CBT and contemporary CBT, named Change Of Recurrence, which aimed to improve the management of FCR in patients with cancer and survivors. We would opt for a blended intervention for 3 reasons. First, given the health emergency that we have been experiencing for the COVID-19 outbreak, a blended intervention would allow us to guarantee the safety of the patient who will rarely have to go to the hospital while maintaining the therapeutic alliance, which is fundamental to the effectiveness of any psychological intervention [81]. Web-based interventions are generally well-received by patients with cancer and might enhance comprehensive care [82], whereas blended therapy overcomes the disadvantages of the lack of alliance between eHealth interventions, maintaining the benefits of a face-to-face intervention [59-61] and the cost reduction and increased accessibility of delivering mental health care [83]. Furthermore, in the retrieved literature, the blended modality resulted as effective in reducing the FCR with moderate-to-large effect sizes, maintained across time [41,47,63,71]. Overall, we propose an intervention based on a mix of the key elements that characterize the effective approaches retrieved in the aforementioned literature (CBT, ACT, and mindfulness), delivered through a combination of face-to-face and remote sessions and psychoeducational material.

The program would be structured by first conducting face-to-face sessions at the hospital where the patient is treated or where the survivor undergoes check-ups. This choice could help patients and survivors follow the therapy, especially if we insert the session on the same day that the patient or survivor goes to the hospital for other visits or checks. Indeed, dropout from psychological interventions is a relevant issue to be considered. In addition, we will create a web-based platform comprising 10 modules that can be accessed only when patients finish the previous one. The web-based platform will provide interactive sessions, psychoeducational exercises, and homework. The latter has the objective of trying to render patients as autonomous as possible by applying the techniques learned in the face-to-face sessions.

#### First Face-to-face Session

During the first face-to-face session, the therapist gets to know the patient, assessing the level of FCR both qualitatively and quantitatively, using the questionnaire Fear of Cancer Recurrence Inventory. In particular, the patient provides details about him or herself and has the opportunity to discuss diagnosis; treatment; recovery; and, in general, his or her experience. Moreover, this will be a psychoeducational session in which the therapist will explain the FCR model, identify the internal and external triggers that increase the FCR, and focus on the patient’s FCR experience, particularly on their maladaptive thoughts and coping strategies. At the end of the session, the therapist will give the patients a link to the web-based platform. Through the platform, the patients will be asked to write their negative automatic thoughts using a typical 3-column grid.

#### First Web-Based Session

In the first web-based module, the patient will find a summary of the key concepts addressed during the first face-to-face session and exercises to do, such as writing down the thoughts and actions that he or she performs and that increase the FCR.

#### Second Face-to-face Session

During the second face-to-face session, the therapist will discuss the patient’s homework to clarify the eventual unsolved aspects. In this way, the patient will be encouraged to share his or her experience and start to work on it. The therapist will then introduce the notion of cognitive restructuring, and through the Socratic questioning of cognitive therapy, attempt to challenge the negative automatic thoughts written by the patient during homework. Finally, the therapist and patient will start to reframe negative thinking into alternative thoughts that are more based on reality to explain the homework that the patient will have to do during the week.

#### Second Web-Based Session

In the second web-based module, the patient will find a summary of the key concepts addressed during the second face-to-face session. The assigned homework that the patient will have to do is to write the negative automatic thoughts using a typical 5-column grid and reframe them into realistic thinking.

#### Third Web-Based Interactive Session

During the third web-based interactive session, the therapist will review the patient’s homework. Then, the therapist and the patient will discuss their thoughts and feelings related to cancer and the actions they will take to get rid of or escape those feelings and those that increase the FCR. In particular, the therapist will provide psychoeducational concepts about worry, explaining the importance of expressing fears. To do that, the patient will have to describe their worst-fear scenario related to cancer, providing thoughts and behaviors that he or she will engage in. Then, the therapist will use metaphors such as the bus metaphor, in which the patient will identify thoughts, feelings, and memories or images as *passengers* persistently challenging cancer. In this way, the patients will learn to actively accept, defuse, and respond compassionately to passengers while not allowing them to dominate their lives. Finally, the therapist will introduce the concept of relaxation, in particular, body scan meditation.

#### Fourth Web-Based Session

Following the third module, the patient will find a summary of the key concepts addressed during the third face-to-face session on his or her platform. The patient will also be provided with other metaphors and experiential exercises (eg, daily body scan meditation) aimed at improving the strategies built in the web-based interactive session with the therapist.

#### Fourth Face-to-face Session

During the fourth face-to-face session, the therapist will focus on adaptive coping strategies that enhance acceptance, cognitive defusion, awareness, and psychological flexibility in general. In this way, the patient will begin to become aware and accept thoughts and emotions about cancer, eliminate rigid thoughts and beliefs about cancer, and define personal values and commit to pursuing meaningful activities in line with those values. Finally, the therapist will introduce the concept of mindfulness and its basic principles.

#### Fifth Web-Based Session

In this web-based module, the patient will be provided with a summary of the key concepts addressed during the fourth face-to-face session, with particular attention to mindfulness. Specifically, the patient will be presented with mindfulness and relaxation exercises through audio and videotape. The audio clips will contain fully automated exercises meant to bring awareness to breathing and bodily sensations. The video clips will provide additional explanations on the techniques, along with some practical guidelines on how to practice during the daytime without clips.

#### Fifth Face-to-face Session

During the last face-to-face session, the therapist will evaluate the changes that occurred in the patient’s emotional and cognitive reactions, making a summary of the current situation and the changes that occurred. The therapist and the patient will talk about the differences between how the patient coped with FCR before the treatment and how he or she copes now. Moreover, the therapist and the patient will draw up an action plan based on the patient’s values. Finally, the therapist will build, together with the patient, an exercise schedule to maintain the improvements. The contents of the exercises (audio or video clips) will be available on patients’ platforms and will be accessible for a year.

#### Follow-up Web-Based Interactive Session

A final web-based interactive session will be provided 1 month after the fifth face-to-face session to monitor the psychological state of the patients.

If effective, this program would lead to a time and cost-saving care pathway for treating FCR, putting together the benefits of real-time interaction with the clinical staff and the ease of having tailored clinical materials available daily to allow for a continuous improvement.

### Conclusions

To conclude, this overview has some limitations. Regarding the first part (review of the psychological interventions), the limitations of the methodology of the included studies and between-study heterogeneity reduced the overall strength of the evidence. Moreover, some of the studies were selected from other systematic reviews, whereas others were selected manually. Regarding the second part, it is only a preprotocol that must be evaluated by experts and patients.

## Abbreviations

ACT: acceptance and commitment therapy
AIM-FBCR: Attention and Interpretation Modification for Fear of Breast Cancer Recurrence
BC: breast cancer
BC-CBT: blended care cognitive behavioral therapy treatment
CBT: cognitive behavioral therapy
eMBCT: internet-delivered mindfulness-based cognitive therapy
FCR: fear of cancer recurrence
MBCT: mindfulness-based cognitive therapy
MBSR: mindfulness-based stress reduction
QoL: quality of life
RCT: randomized controlled trial
